# Towards a Formal Context-Aware Workflow Model for Ambient Environment

**DOI:** 10.1007/978-3-030-51517-1_38

**Published:** 2020-05-31

**Authors:** Roumeissa Khennaoui, Nabil Belala

**Affiliations:** 8 Digital Research Centre of Sfax, Sfax, Tunisia; 9 Institut Mines-Télécom, CNRS, Paris, France; 10 Université de Sherbrooke, Sherbrooke, QC Canada; 11 Digital Research Centre of Sfax, Sfax, Tunisia; 12 University of Sfax, Sfax, Tunisia; MISC Laboratory, University of Constantine 2 Abdelhamid Mehri, Constantine, Algeria

**Keywords:** Modeling of physical and conceptual information in smart environments, Context awareness, Ambient intelligence, Formal description, Workflow

## Abstract

Ambient systems owns some particular characteristics that makes their context awareness a sincere problem; they are composed of heterogeneous distributed devices, some of these devices may appear and disappear during operations. In addition, users interacting in these systems are themselves dynamic. Therefore, context-aware workflow management allows workflows to adapt dynamically according to the environment changes. Context information are complex and diverse which makes the modeling the key issue. This paper presents an approach to model context-aware workflows. First, we describe the workflow using Ag-LOTOS. Then, based on this description, we build the contextual planning system *CPSw* that allows the presentation of the context at each activity state.

## Introduction

In order to meet the needs of everyday life, systems are becoming more and more complex, which leads to seek to give more atomicity and initiative to the different software modules. To respond to this technological evolution, ambient intelligence [1] is a new paradigm of distributed systems where the environment is aware of the user’s needs and find a way to fulfill that need to improve the quality of people’s life.

Due to the extreme mobility of users, ubiquitous software [2] run in a highly dynamic and varying environment. Therefore, context awareness [3] and context adaptation [4] are some important aspects for pervasive software that have to be aware of the context’s changes, and dynamically adjust their execution [4]. Context-aware workflow [5] is an interesting field that allows workflows to adapt dynamically to the context changes in ubiquitous environment.

To achieve this goal, no many results have been accomplished in workflow’s context modelling. [6] proposes a context-aware workflow management system (WFMS) for navigation applications in ubiquitous computing. In [7], a dynamic context-aware access control for pervasive computing in enterprise environment is proposed. However, [8, 9] allow users to model their daily activities in the form of workflow adaptable to context information. [10] proposes an approach to build a flexible model to adapt business process based on context. In [11], both the conceptual model and the workflow model are defined based on OWL.

Considering that the ambient systems manage our daily life such as smart hospital, smart home, robots, etc., errors are critical regarding human life. Many WFMS tools [12, 13] exist and allow the modelling and verification of workflows. However, mathematical approaches are proved to be more effective [14]. [15] describes the workflow patterns in the formal specification language LOTOS [16]. In addition, [17] proposes an approach to specify and verify the service composition using LOTOS.

In this paper, we describe at first the workflow using Ag-LOTOS [18], a formal specification model based on LOTOS. Ag-LOTOS is a formal technique based on process algebra that allows to formally describe the workflow and to verify properties on the model. Then, the contextual planning system of the workflow (CPSw) [19] is built based on the semantics of Ag-LOTOS constrained by contextual information. Unlike the previous work, our approach allows a formal description of the context as *pre*- and *post*-condition in each state of all the possible traces and adjust the changes dynamically. The proposed model can be used in the verification process to check some contextual properties.

## The Context-Aware Workflow Model

### Ag-LOTOS for Workflows

Business Process Management (BPM) has been defined by van der Alast as “a way to support business processes using methods, technique and software to model, execute, control and analyze operational processes involving humans, organizations, applications, documents or any other source of information” [20]. However, a workflow can be defined as “business process automation during which documents, information or tasks are passed from one participant to another according to a set of process rules” [20]. A workflow pattern [21] represents the abstraction of most frequent activities sequence, and are composed when specifying new workflows.

In this section, we aim to use Ag-LOTOS [18] to improve workflow specification by including contextual information to each state of the workflow model, and by modeling the ambient characteristics such as communication and mobility that cause the dynamic changes of the context.

Similar to LOTOS [16], Ag-LOTOS concurrency allows the modeling of parallel activities. The Ag-LOTOS subsystem support allows the composition of workflow elements. Since Ag LOTOS is derived from LOTOS, we follow the procedure of mapping of the workflow patterns to LOTOS notation applied in [14] and citecite6 to give the suitable definition of each operator in the workflow context. To specify activities in details, we can simply model them with Ag-LOTOS sub-processes (hierarchy of processes). Note that Ag-LOTOS processes are used to model activities in the workflow. However, the process in the workflow context indicates a set of activities.

Ag-LOTOS expressions are written by composing actions through the LOTOS operators.

The syntax is defined as follows [18]:

$$\begin{aligned} \quad a;E | E\odot E \quad (a\in \partial ) \end{aligned}$$

$$\begin{aligned} |hide \, L \, in \, E \end{aligned}$$

$$\begin{aligned} |x!(v) |x?(v) \quad (x \in \mathcal {U} , v \in \mathcal {M} ) \end{aligned}$$

Where $\partial$ is a finite set of observable actions, L is a subset of $\partial$ and $\mathcal {H} \subset \partial$ is the set of ambient intelligence primitives, which represent the mobility and the communication. $\ominus$ is the finite set of spatial localities of the pervasive environment, $\mathcal {U}$ is a finite or infinite set of users, with which the user can communicate, and $\mathcal {M}$ is the set of messages that can be sent or received.

An essential component of a process definition is its behavior expression E. A behavior expression is built by applying an operator, e.g., $\gg$, to other behavior expressions. A behavior expression may also include instantiations of other processes.

Termination.: In Ag-LOTOS, the termination is represented via the operator *stop* witch indicates the inaction while the *exit* operator expresses the successful termination.
Fail.: In $A={fail}$, *fail* represents the fact that the execution of an activity *A* fails because of the dynamic context of the workflow.
Prefix.: The operator ‘;’ is used to prefix a behavior expression with an action to produce a new one. Note that actions are the elementary units executed by activities.
Hiding.: *hide* is used to express the discriminator pattern (similar to LOTOS). An external gate is used to invoke the subprocess that enables the activity. This gate is hidden inside the discriminator to avoid any external synchronization (see [14, 15] for further details).

Respectively, the set $\odot$ represents the standard LOTOS operators.

Sequence.: The sequential composition operator $\gg$ is used to represent the sequence pattern.
Cycle.: A loop in a process allows the repetitive execution of activities, P.
Choice.: $A\,[\,]\,B$, activity *A* or *B* will be chosen.
Disabling.: During the activity execution, it is possible to indicate its failure with the disabling operator $[>$. $A\,[>B$ means activity *A* may be disabled by activity *B* which interrupts the main flow and uses *stop* instead of *exit*.
Parallelism (general case).: $A\,|[L]|\,B$ means if the process (activity *A*) is ready to execute some action at one of the synchronization gates, it is forced, in the absence of alternative actions, to wait until the process (activity *B*) offers the same action.
Full Synchronization.: $A \parallel B$ means that if $L = \partial$, the two composed activities are forced to execute in complete synchronicity.
Pure Interleaving.: If $L= \emptyset$, the absence of synchronization leads to the absence of interaction points among processes, this is achieved through the interleaving operator ‘|||’.

### Contextual Planning System of the Workflow

In order to illustrate the concept of the formal design of workflows with the contextual information, the contextual planning system is built from an Ag-LOTOS specification using the rules in Table 1.

**Table 1.** The semantic rules.

Action:		$\dfrac{ ws \xrightarrow {a} ws' \quad a \in Act}{(ws,l) \xrightarrow {a} (ws',l)}$
Mobility:		$\dfrac{ ws \xrightarrow {move(l')} ws' \quad (l \ne l')}{(ws,l) \xrightarrow {move(l')} (ws',l')}$
Communication:		$(a) \dfrac{ ws \xrightarrow {x!(u)} ws' \quad (u \in \mathcal {U} )}{(ws,l) \xrightarrow {x!(u)} (ws',l)} \quad (b) \dfrac{ ws \xrightarrow {x?(u)} ws' \quad (u \in \mathcal {U} )}{(ws,l) \xrightarrow {x?(u)} (ws',l)}$

The Contextual Planning System of the Workflow (CPSw) based on CPS [22] takes into account two types of information: workflow planning state *ws* and locality *l*. Table 1 shows the operational semantic rules that define the possible planning state changes for the workflow. From an initial planning state ($$ ws_{0} ,l$$), we apply these rules to produce the CPSw. The contextual planning system CPSw is a labeled Kripke structure $$(S, s_{0}, Tr , L)$$ where *S* is the set of contextual planning workflow states, $$ s_{0} = ( ws_{0} , l)\in S$$ is the initial planning state of the workflow, $$Tr\subseteq S \times \partial \cup \{T\} \times S$$ is the set of transitions which are denoted $$ s \xrightarrow {a} s^{\prime }$$, and $$L:S \rightarrow \ominus $$ is the location labeling function.

## Case Study

In this paper, we target on context-aware workflow models for ubiquitous company. Let there be an enterprise with several helpdesk employees associated with smart badges that provide the system with spatial information at each moment.

**Fig. 1.**
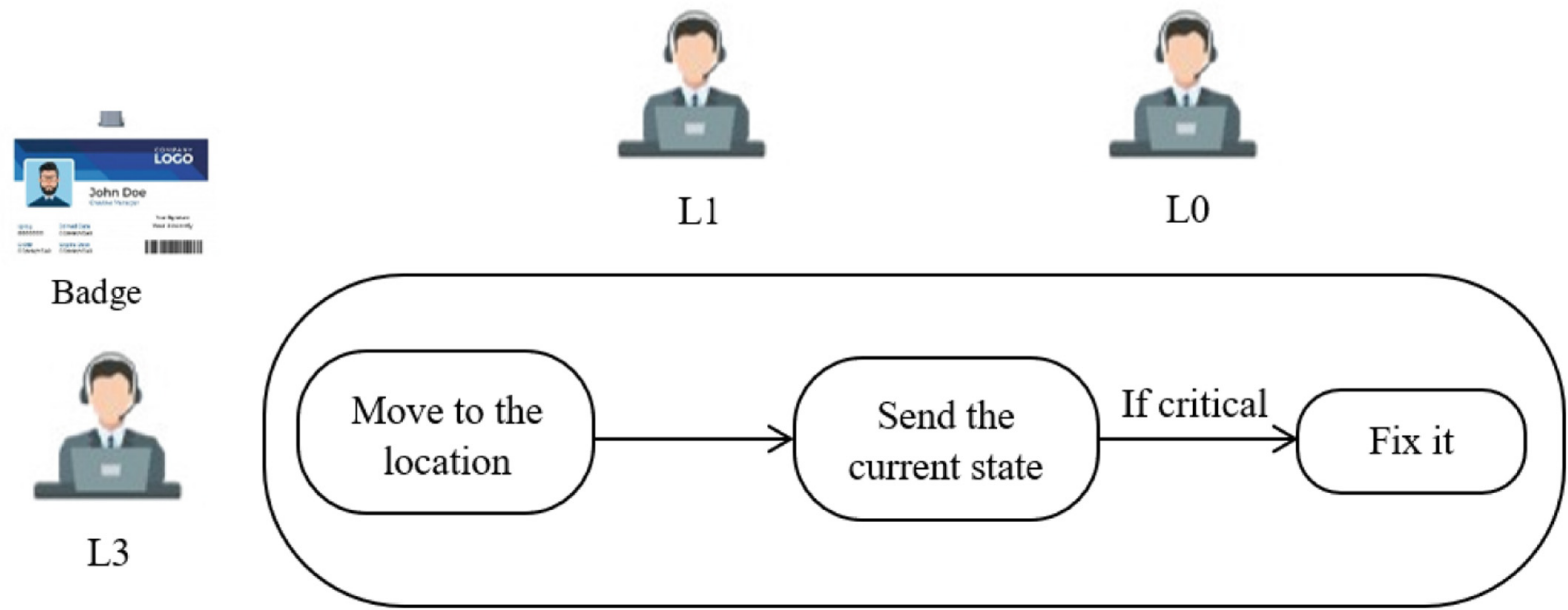
The scenario that illustrate the case study.

The scenario illustrated in Fig. 1 is highly context dependent, especially in the following way:

**Fig. 2.**
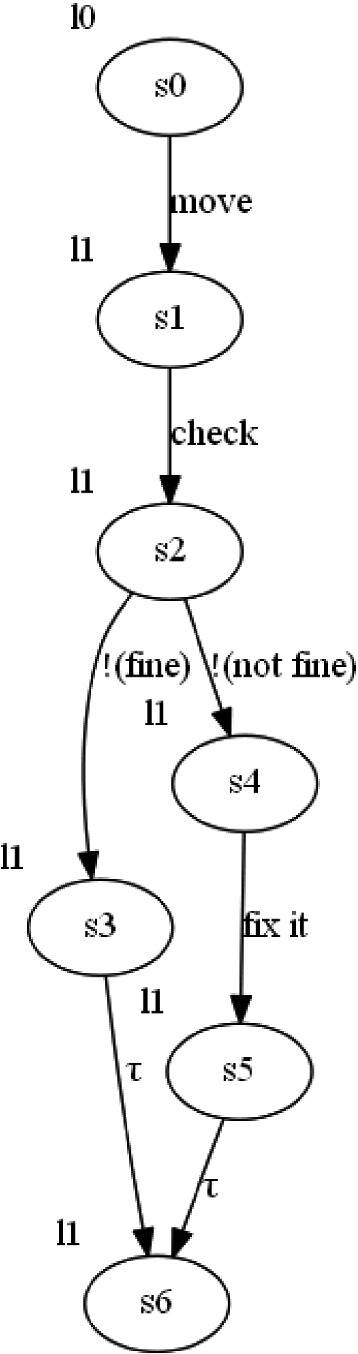
The CPSw corresponding tot the case study.

If the system detects a problem on the switch in datacenter location, he sends a request to the closest helpdesk. This one has to move to the location of the switch and send the current state by email to the management system:

State 1: fine, indicate that there is no critical problem.
State 2: not_fine, in case of critical one which need to be fixed.

The corresponding CPSw to the scenario is illustrated in Fig. 2. It is built from the initial Ag-LOTOS description:
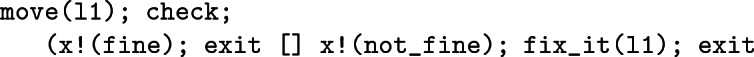


## Conclusion

Workflow systems are currently used by many organizations including health care, automation and finance. Context awareness is the ability for workflows to react to the changing situations. In this paper, we introduced a context-aware workflow model, the CPSw, that presents all the possible evolutions of workflow’s activities constrained by the contextual information. CPSw is constructed formally based on Ag-LOTOS description giving the set of activities.

We learned that using Ag-LOTOS to describe workflow activities is a promising approach. Mainly, because it allows a formal description of the current context in each state as *pre*- and *post*-conditions, and dynamically adjusts the modifications. Furthermore, it allows the verification and validation of the model.

The proposed model can be used in the verification process to verify certain contextual properties. For future works, we aim to consider different types of context information such as the time.
